# Indoor Temperatures in the 2018 Heat Wave in Quebec, Canada: Exploratory Study Using Ecobee Smart Thermostats

**DOI:** 10.2196/34104

**Published:** 2022-05-12

**Authors:** Arlene Oetomo, Niloofar Jalali, Paula Dornhofer Paro Costa, Plinio Pelegrini Morita

**Affiliations:** 1 School of Public Health Sciences Faculty of Health University of Waterloo Waterloo, ON Canada; 2 Department of Computer Engineering and Automation School of Electrical and Computer Engineering University of Campinas Campinas Brazil; 3 Institute of Health Policy, Management, and Evaluation University of Toronto Toronto, ON Canada; 4 Department of Systems Design Engineering University of Waterloo Waterloo, ON Canada; 5 Research Institute for Aging University of Waterloo Waterloo, ON Canada; 6 eHealth Innovation Techna Institute University Health Network Toronto, ON Canada

**Keywords:** Internet of Things, IoT, heat waves, public health, smart home technology, smart thermostats, indoor temperature, air conditioning, heat alert response systems, thermostat, unsafe temperatures, uHealth

## Abstract

**Background:**

Climate change, driven by human activity, is rapidly changing our environment and posing an increased risk to human health. Local governments must adapt their cities and prepare for increased periods of extreme heat and ensure that marginalized populations do not suffer detrimental health outcomes. Heat warnings traditionally rely on outdoor temperature data which may not reflect indoor temperatures experienced by individuals. Smart thermostats could be a novel and highly scalable data source for heat wave monitoring.

**Objective:**

The objective of this study was to explore whether smart thermostats can be used to measure indoor temperature during a heat wave and identify houses experiencing indoor temperatures above 26°C.

**Methods:**

We used secondary data—indoor temperature data recorded by ecobee smart thermostats during the Quebec heat waves of 2018 that claimed 66 lives, outdoor temperature data from Environment Canada weather stations, and indoor temperature data from 768 Quebec households. We performed descriptive statistical analyses to compare indoor temperatures differences between air conditioned and non–air conditioned houses in Montreal, Gatineau, and surrounding areas from June 1 to August 31, 2018.

**Results:**

There were significant differences in indoor temperature between houses with and without air conditioning on both heat wave and non–heat wave days (*P*<.001). Households without air conditioning consistently recorded daily temperatures above common indoor temperature standards. High indoor temperatures persisted for an average of 4 hours per day in non–air conditioned houses.

**Conclusions:**

Our findings were consistent with current literature on building warming and heat retention during heat waves, which contribute to increased risk of heat-related illnesses. Indoor temperatures can be captured continuously using smart thermostats across a large population. When integrated with local heat health action plans, these data could be used to strengthen existing heat alert response systems and enhance emergency medical service responses.

## Introduction

### Background

Our planet is getting warmer, and climate experts report that the frequency of extreme heat days has been increasing—record-setting temperatures have been reported in major cities around the world [1]. Driven by the output from human activities (such as the greenhouse effect from gas emissions), the frequency and duration of extreme heat events are only expected to increase [2].

In the United States, heat waves kill more people than all other weather-related events (earthquakes, tornadoes, hurricanes) annually [3]. Periods of extreme heat are dangerous because heat stress occurs when the human body is unable to cool itself [4]. Heat waves in Europe in 2003 and Russia in 2010 caused over 100,000 deaths combined [5]. In 2018, Quebec, Canada experienced a severe heat wave that resulted in 66 deaths [6].

Older adults, persons with disabilities, and persons with chronic conditions such as respiratory diseases and heart disease are more vulnerable during extreme heat days due to their impaired ability to regulate body temperature [7,8]. Individuals with low sociodemographic status are at a higher risk for suffering the detrimental and even deadly effects of a heat wave [9] because cooling methods may be cost-prohibitive [10] or they may live in older buildings without air conditioning units. Those living in urban areas are at greater risk for extreme heat events. Urban areas are often several degrees hotter than rural areas [11]. Densely populated urban areas experience *heat domes*, wherein the built environment absorbs and traps heat instead of reflecting it into the atmosphere [12-15]. Even at night, the temperatures remain high because building materials (such as concrete and asphalt) continue to radiate heat [16-19].

To prevent heat-related deaths, many governments have adopted heat health action plans that include ensuring residents are appropriately informed, providing resources such as cooling centers, and door-to-door checks by emergency services [17]. Heat health action plans are localized and specific to each municipality, tailored to local needs [2,18,20]. These action plans rely upon heat alert and response systems [21] or heat health warning systems. Weather forecasts, based on outdoor meteorological data, are used to predict weather conditions that could be potentially hazardous to health [22].

There are no universal criteria used for issuing a heat wave warning [23]. Environment and Climate Change Canada issues heat warnings for each province. For example, in Quebec, a heat warning is issued when (1) the temperature is 30°C or higher, with a humidex value of 40 or higher for at least one hour, or (2) when the temperature is 40°C or higher [22]. Local health departments also issue extreme heat alerts and often use different criteria. *Santé Montréal* [24], a local health department, defines an extreme heat episode as either 3 consecutive days when the average maximum temperature reaches 33°C and the average minimum temperature does not drop below 20°C or when the temperature does not drop below 25°C for 2 consecutive nights.

Current heat alert and response systems rely upon outdoor meteorological data, yet individuals now spend most of their time indoors [25]. Indoor temperatures during heat waves can be significantly higher than outdoor temperatures [26]. There are discrepancies between temperature data being used to make decisions about heat waves and temperatures actually experienced indoors by individuals. Our understanding of indoor temperature trends is relatively limited because studies on indoor environments can be challenging, time-consuming, and cost-prohibitive due to factors such as sensor costs, cost of study deployment and duration, resources, and disturbances to home life. Traditional studies on heat waves and urban heat islands typically use satellite imagery data [27,28], local or airport weather stations [29], or emergency room visit, mortality, and ambulance call data [30] to set thresholds or examine outdoor heat exposure risk. Only a few studies have investigated indoor temperature related to mortality and morbidity [31] or indoor temperature exposure [32,33].

The advent of smart home technology (ie, indoor temperature sensors) coupled with the Internet of Things (IoT) offers a unique opportunity to monitor residential indoor environments across a large population. IoT sensors have been successfully used to monitor indoor health behaviors including sleep [34,35], gait [36], breathing, and heart rate [37]. Smart thermostat adoption has been driven by government incentives, such as the Ontario Government’s Green Ontario Fund, and a desire save on heating and cooling costs [38,39].

We searched Scopus and Google Scholar, with no language restrictions, for publications since database inception until December 31, 2020, using the search string *(“smart home thermostat” OR “smart thermostat”) AND (“heatwave” OR “heat wave”) OR (“public health”)*. We identified 2 studies [34,35] that used smart thermostats for public health surveillance, with focuses on healthy behaviors such as sleep, physical activity and sedentary behavior. One study [34] validated the use of smart thermostats for measuring sleep, physical activity, and sedentary behavior and found results to be highly comparable with results captured through traditional survey methods used by the Public Health Agency of Canada. The other study [35] found that using smart thermostats allowed for insights into differences (which were significant) in time spent indoors during the weekend versus during weekdays. Both studies [34,35] demonstrated that smart thermostats and remote motion sensors can be used with minimal interference to individual to monitor activity levels routine in real-world settings (ie, in the home).

To the best of our knowledge, this is one of the first studies to use smart home thermostat data to investigate the effects of heat waves. As heat waves increase in frequency, intensity, and duration, the use of indoor cooling methods such as air conditioning will increase. The use of smart thermostats to capture indoor temperature data has several benefits, such as minimal disturbance to study participants, and overcomes barriers associated with many environmental data collection studies (ie, high overhead costs, equipment costs, small sample sizes, short study durations). In many cases, temperature, motion, and humidity data are already being collected. This overlooked source of indoor temperature can be used to strengthen public health response and climate mitigation efforts for decision-making during extreme heat events.

### Objective

We aimed to compare indoor temperatures during heat wave and non–heat wave periods between air conditioned and non–air conditioned houses with ecobee smart thermostats and identify extreme indoor temperatures posing a health risk.

## Methods

### Data Collection

We used smart home thermostat data (collected by ecobee and made available to researchers through the Donate Your Data program [40]) and Environment and Climate Change Canada [41,42] data from in Quebec, Canada between June 1 and August 31, 2018, which included a multiday heat wave event. Outdoor temperatures at weather stations were obtained from Environment and Climate using an amended Python3 script [42,43]. Indoor temperature and occupancy data had been collected via the smart thermostat and remote motion sensors at 5-minute intervals.

### Smart Thermostat Data Processing

Thermostat metadata (number of sensors in a home, HVair conditioning mode setting, and household ID) and indoor temperature timeseries data were available from a total of 768 households. The metadata file also contained information used to associate the house with the nearest weather station. We calculated the mean indoor temperature for each house each hour and each day.

For each day in the period from June 1 to August 31, houses were labeled as non–air conditioned if the cooling stage status was 0 and *cool* was not recorded for *HVair conditioning mode* in the metadata, and houses were labeled as air conditioned if the cooling stage status was 1, 2, 3, or 4 and *HVair conditioning mode* was *cool* at any point during the day. Data from days for which a given house was unoccupied were removed.

To determine whether non–air conditioned houses were more likely than air conditioned houses to have elevated indoor temperatures, we focused on a subset of 82 non–air conditioned households and 96 air conditioned households in the areas of greater Montreal and Outaouais. This was done after data cleaning, remove of incomplete data and filtering for occupancy (determined by activation of motion sensors).

### Mapping Indoor to Outdoor Data

Each house was assigned to the nearest Environment Canada weather station using thermostat metadata. Houses with incomplete data were removed. Three weather stations—Ottawa Gatineau, Montreal/St Hubert, Montreal Intl A—were selected for further study based on having a minimum of 30 houses with data within their associated region and being within a region for which a heat wave had been declared (Multimedia Appendix 1). Montreal and Outaouais (Gatineau and surrounding areas) experienced a 6-day heat wave (from June 30 to July 5, 2018) [41].

### Data Analysis

We developed and used an app (RShiny) to visualize and compare indoor and outdoor temperature trends.

We compared the indoor temperatures of non–air conditioned houses during heat wave and non–heat wave days. After combining all the daily records from each group, we used *t* values to examine temperature differences between air conditioned and non–air conditioned houses.

A 1-tailed *t* test (with unknown variance) was performed to comparing the indoor temperatures of all houses in the same region, with the assumption that they experienced the same outdoor temperature during extreme heat events and that the indoor temperatures for non–air conditioned households was consistent.

Literature indicates that 26°C (72°F) is the threshold for a safe indoor temperature (ASHRAE 55 indoor temperature standard [44]). Exposure to indoor temperatures greater than 26°C has been associated with increased premature mortality and emergency medical service calls [45]. We sought to determine whether above-threshold indoor temperatures were recorded during the heat wave in regions of Quebec (Montreal and Gatineau) for which heat waves had been recorded on specific dates [41,46]. Remaining households were further filtered to remove those not in areas where heat waves were officially declared so that analysis contained only houses that experienced the heat wave. Thus 47 non–air conditioned homes from Montreal, Laval, Montérégie, and Outaouais health regions of Quebec remained after removing households that used air conditioning between June 1 and August 31, 2018 and houses with no occupancy on heat wave days (who may have turned off air conditioning while away). To examine which time of day the highest indoor temperatures occurred, we created a heat map of the non–air conditioned home temperatures for each hour on each official heat wave day.

## Results

**Figure 1:**
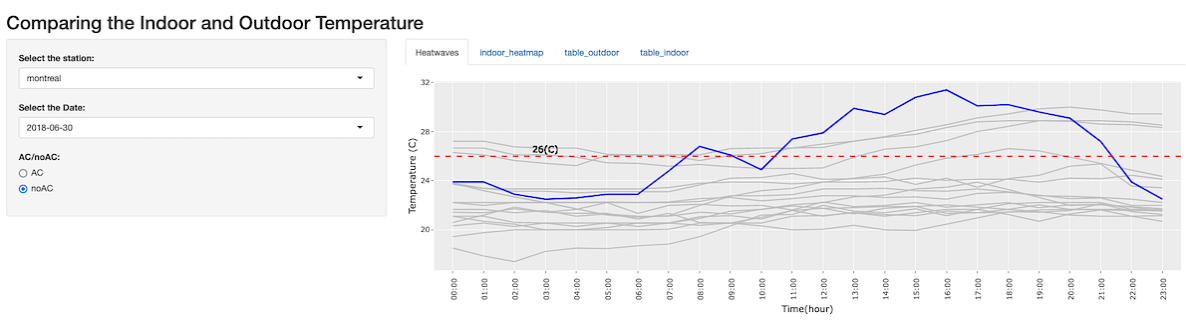
Screenshot from the RShiny app, which shows a comparison of indoor (gray lines) and outdoor (blue line) temperatures for non–air conditioned houses in the Montreal/St. Hubert weather station region on June 30, 2018.

On certain days, some non–air conditioned homes recorded indoor temperatures similar to, or even exceeding, the outdoor temperature on heat wave dates (Figure 1). These results demonstrate that using IoT devices such as the ecobee smart home thermostats are a logical means of monitoring indoor residential temperatures, especially during extreme heat events.

### Indoor Temperature Differences Between Air Conditioned and Non–air conditioned Houses

There is a significant difference of indoor temperature between the houses with air conditioning and without air conditioning in heat wave and non–heat wave days (*P*<.001). On heat wave days, the average daily indoor temperature was lower (mean 1.3°C) for air conditioned homes than that for non–air conditioned homes. This difference is lower in non–heat wave days (mean 0.5°C) (Table 1).

Indoor temperatures of houses with air conditioning during the heat wave days and non–heat wave days remained relatively stable, with mean temperatures 22.3°C and 22.4°C for non–heat wave and heat wave days, respectively.

For houses without air conditioning, there was a statistically significant difference (*P*<.001) between indoor temperatures on heat wave days (mean 23.7°C) and non–heat wave days (mean 22.8°C), which were, on average, 0.90°C hotter (Table 1).

There were significant differences in indoor temperatures between houses with and without air conditioning for all 3 regions (all *P*<.001).

**Table 1:** Indoor temperature comparisons.

					Mean difference (°C)			Standard difference (lower, upper) or 99% CI			*P* value
**Air conditioned vs non–air conditioned houses**											
		During heat wave days^a^	–1.320			–0.730 (–1.90, –0.73)			<.001		
		During non–heat wave days^b^	–0.500			–0.021 (–0.615, –0.40)			<.001		
**Non–air conditioned houses only**											
	Heat wave vs no heat wave^c^			0.906			0.784 (0.350, 1.45)			<.001	

^a^Air conditioned: n=465; non–airconditioned: n=151.

^b^Air conditioned: n=6045; non–airconditioned: n=2713.

^c^Non–air conditioned—during heat wave: n=151; non–heat wave: n=2713.

### Non–air conditioned Houses With Indoor Temperatures Above Safe Thresholds in Health Regions With Heat Waves

**Figure 2:**
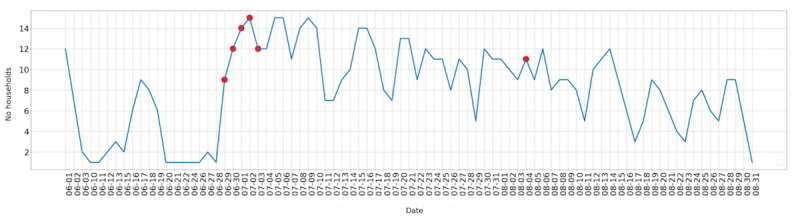
The number of households that experienced indoor temperatures equal to or greater than 26ºC for each date. Official heat wave days indicated by a red dot.

Figure 2 shows the number of non–air conditioned houses, out of 47 located in regions with recorded heat waves, that experienced a daily temperature of or above 26°C between June 1 and August 31. There were consistently a number of non–air conditioned households with temperatures of or above 26°C, on heat wave days as well as non–heat wave days, especially during July and August.

**Figure 3:**
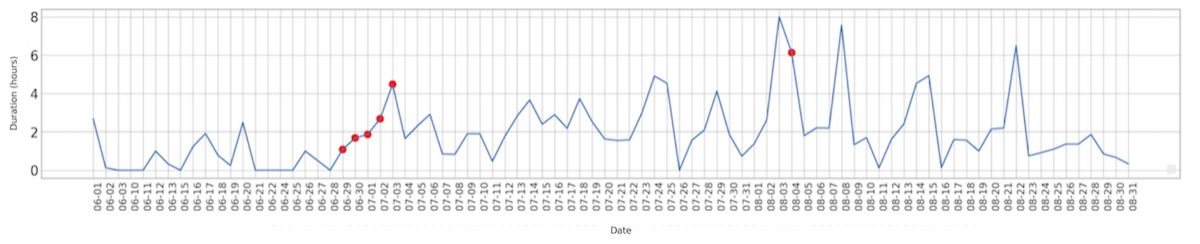
The average duration (in hours) with indoor temperature equal to or greater than 26 ºC for houses without air conditioning. Official heat wave days indicated by a red dot.

Figure 3 illustrates the average duration (in hours) that the 47 non–air conditioned households recorded indoor temperatures to equal to or greater than 26°C from June 1 to August 31.

**Figure 4:**
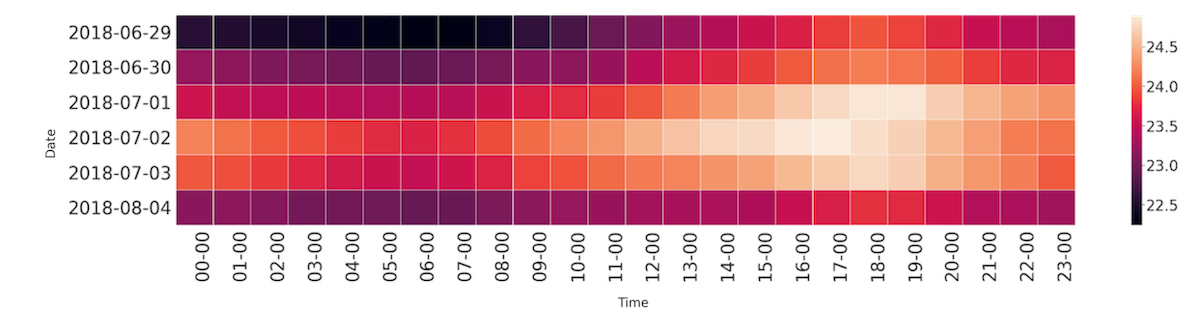
Average indoor temperatures for 47 households on heat wave days over time.

Indoor temperatures were highest during the afternoon, evening, and night-time periods of the day (Figure 4); the hottest time of the day was between 4 PM and 7 PM on heat wave days. During the 5-day heat wave from June 29 to July 3, it is possible to see the accumulation of residual heat later into the evening and into the early hours of the day.

## Discussion

### Principal Findings

There were high indoor temperatures for prolonged periods of time that may put people at risk; indoor temperatures may be important to consider in policies and heat mitigation strategies. In Quebec, current heat alert and response systems rely upon outdoor meteorological data, which do not reflect actual indoor temperatures experienced by individuals. There were statically significant differences between air conditioned and non–air conditioned houses: non–air conditioned homes experienced a greater than 1°C difference in temperature between heat wave and non–heat wave days. Because there were only 6 official heat wave dates during the summer of 2018, comparison dates were limited. We also found a statistically significant difference between indoor temperatures for non–air conditioned houses and air conditioned homes during a heat wave (*P*<.001). Additionally, we found that there were significant differences between indoor and outdoor temperatures across all households (*P*<.001).

During the summer of 2018, there were numerous non–air conditioned houses that experienced indoor temperatures greater than the ASHRAE 55 standard of 26°C for extended periods of time. This indoor temperature standard [47], which is used widely in spaces such as office buildings, does not take into account factors such as age or health. The threshold was exceeded both on official heat wave days and on non–heat wave days. We also observed that the hottest time of the day indoors was between 4 PM and 7 PM, with increases in temperature over the 5-day heat wave.

We were specifically interested in indoor temperatures recorded during the official heat wave from June 30 to July 5, 2018. Unsurprisingly, the indoor temperatures of homes with air conditioning remained relatively stable (mean 22.3°C or approximately 72°F) with no dramatic temperature spikes. Some air conditioned homes did record higher indoor temperatures. This may be due to homeowners having programmed the thermostat to maintain a higher indoor temperature to save on energy costs. Several non–air conditioned dwellings with ecobee thermostats had indoor temperature readings between 27°C (81°F) and 32°C (90°F), which is above the safe indoor threshold of 26°C (79°F) that is recommended by numerous health policy documents [44,45,48]. Smart home technology was able to capture continuous temperature data, which allowed the identification of houses that had experienced higher than ideal indoor temperatures. This technology could allow local governments to develop hyperlocal, real-time heat alert and response systems to protect health [49].

On official heat wave days, many homes recorded indoor temperatures reaching 26°C. Temperatures as high as 32.7°C were recorded over multiple hours in several households on heat wave days. This information is important considering that spending more than a few hours indoors in a hot room can be hazardous to health [47]. Non–air conditioned homes experienced indoor temperatures above 26°C for an average of 4 hours; there was an upward trend in duration as the heat wave persisted. A second official heat wave event was declared on August 4 in the Outaouais health region. In this region, homes without air conditioning experienced indoor temperatures for more than 9 hours above 26°C. Prolonged periods with indoor temperatures above 26°C were also experienced by non–air conditioned dwellings on unofficial heat wave days (July 14, July 23, July 31 to August 3, August 7, August 11 to August 15). In fact, houses in the Montreal and Outaouais areas consistently recorded indoor temperatures above 26°C throughout the summer of 2018. While definitions of extreme heat vary by region, heat warnings are issued in Canada when temperatures of 30°C or higher are expected for at least one hour [50]. Consistent with the findings of previous literature [51,52], our findings showed that higher indoor temperatures were reached in the late afternoon, and the coolest temperatures typically occurred in the early morning. Our results emphasize that outdoor temperature is not always a good indicator of safe indoor temperature; thus, there is a need to include indoor temperature monitoring capacity in our public health units.

We plan to explore indoor humidity data in the future. While the humidex is not widely used and critiqued by many, indoor humidity plays a role in human comfort especially during extreme heat events [26,49,53].

There is still relatively little known about indoor temperatures; the ability to study a large number of dwellings is both costly and time-consuming. This study is one of the first to use indoor smart thermostat data to investigate extreme heat events in Canada.

### Strengths and Limitations

Our ability to identify at-risk households has implications for the delivery of emergency services. While in this study, data were not collected in real time, the technology can be used for real-time alerts. For example, the data can be integrated directly with real-time temperature updates to ensure caregivers, community care organizations, emergency medical response, paramedic, or hospital teams can reach at-risk populations. These data can also be used to dispatch emergency medical services to respond to calls and improve the safety of those aging in place or vulnerable individuals who may require alternate care levels. Furthermore, smart thermostats can be used to replace existing thermostats and bring energy and cost savings to home owners while meeting moral, pro-environment values and low-carbon targets [54]. Leveraging smart thermostats instead of introducing new technology could mean less barriers to adoption and easier integration to support heat health warning systems. The volume of data available is another strength of this technology. Although we focused on a short timeframe in a single province, there are still hundreds of datapoints available from location across Canada.

There are currently several limitations to the use of ecobee data to study indoor temperatures. The number of occupants is unknown. In addition, the location of the residence is limited to the city level. This made grouping and matching homes with outdoor weather stations a challenge. This limitation protects anonymity and encourages voluntary enrollment in ecobee's Donate Your Data program [40]. We were unable to account for technology malfunctions, such as a dead battery in a remote sensor, internet disconnection, or a power outage, that may have resulted in missing data. The location of the smart thermostat is entered in a text field by the user during setup, thus, could be inaccurate. Another limitation is that we do not know if other cooling methods, such as a fan or window air conditioning unit, were used in the home. This could explain why some non–air conditioned dwellings experienced stable indoor temperatures. Furthermore, smart thermostats are more likely to be in higher socioeconomic status households, in detached homes that are owned by the resident [55].

### Implications

A greater understanding of indoor temperature is necessary, given the risks of high temperature to human health [44] and the rise in extreme heat events over recent decades [56]. Many factors can contribute to increased indoor temperatures, including the surrounding environment and building materials [57,58]. Canada has put in place some policies to address urban heat, such as planning that include green urban areas with trees and living roofs, encouraging the use of materials that reflect heat into the atmosphere, and retrofitting old buildings with smart, energy-efficient technologies [13].

These results also have implications for schools, hospitals, and long-term care facilities looking to prepare for future decades of warmer temperatures [17,59,60]. We must consider and ensure safe indoor temperatures, particularly for at-risk populations [47,61-63]. Preparation for extreme challenges is vital (ie, lockdowns due to a pandemic). During the COVID-19 pandemic, many public spaces traditionally used for cooling centers (ie, libraries and community centers) were closed, and previous strategies (ie, visiting an indoor mall) were not feasible when public gathering was prohibited due to social distancing measures [64,65].

Our study demonstrates the use of smart thermostat data for heat wave monitoring. The adoption of smart home devices, such as smart thermostats, can be used for a greater purpose and have public health benefits. This technology can be used to build on existing public health heat adaptation interventions and programs; for example, the strategic placement of sensors across neighborhoods can help cities understand how heat affects their citizens. The use of secondary data overcomes some of the challenges associated with a traditionally environmental study. Further research is needed to understand better indoor temperatures in low-income housing or in institutions, such as long-term care and hospitals, that house individuals who are less able to cope with extreme heat and more vulnerable to its effects.

## Appendix

Multimedia Appendix 1 Weather stations in Quebec 2018 and number of associated houses with ecobee smart thermostats.

## Abbreviations

ASHRAE: American Society of Heating, Refrigerating and Air-Conditioning Engineers
IoT: Internet of Things

## Data Availability

Readers may contact the authors for more information on the RShiny app [66]. The code used for the app is available upon request to the corresponding author. The ecobee data are available by reaching out to ecobee directly to become an affiliated researcher.
