# Supplementary material for: Indoor Temperatures in the 2018 Heat Wave in Quebec, Canada: Exploratory Study Using Ecobee Smart Thermostats
Source: JMIR Form Res. 2022 May 12;6(5):e34104. doi: 10.2196/34104 (PMC9136646; doi:10.2196/34104)
Supplement: Multimedia Appendix 1 [file formative_v6i5e34104_app1.docx]

### Appendix 1: Weather station and number of associated ecobee households in Quebec 2018

| **Weather Station** | **No of Households** |
| --- | --- |
| BEAUPORT | 23 |
| GRANBY | 8 |
| ILES DE LA MADELEINE A | 2 |
| JONQUIERE | 5 |
| L'ACADIE | 2 |
| L'ASSOMPTION | 41 |
| LAC BENOIT | 208 |
| LAC MEMPHREMAGOG | 6 |
| LENNOXVILLE | 3 |
| MANIWAKI AIRPORT | 1 |
| MANIWAKI UA | 1 |
| MCTAVISH | 149 |
| MONTREAL INTL A | 70 |
| MONTREAL MIRABEL INTL A | 26 |
| MONTREAL/ST-HUBERT | 80 |
| OTTAWA GATINEAU A | 59 |
| SAINT-GERMAIN-DE-GRANTHAM | 3 |
| SHAWINIGAN | 1 |
| ST-ANICET 1 | 2 |
| ST-JOVITE | 1 |
| STE-ANNE-DE-BELLEVUE 1 | 31 |
| STE-CLOTHILDE | 1 |
| THETFORD MINES RCS | 2 |
| VALCARTIER A | 3 |
